# Comparative Transcriptome Analysis Reveals the Gene Expression and Regulatory Characteristics of Broad-Spectrum Immunity to Leaf Rust in a Wheat–*Agropyron cristatum* 2P Addition Line

**DOI:** 10.3390/ijms23137370

**Published:** 2022-07-01

**Authors:** Xiajie Ji, Taiguo Liu, Shirui Xu, Zongyao Wang, Haiming Han, Shenghui Zhou, Baojin Guo, Jinpeng Zhang, Xinming Yang, Xiuquan Li, Lihui Li, Weihua Liu

**Affiliations:** 1 National Key Facility for Crop Gene Resources and Genetic Improvement, Institute of Crop Sciences, Chinese Academy of Agricultural Sciences, Beijing 100081, China; jixiajie003@126.com (X.J.); xushirui1105@163.com (S.X.); w18731255281@163.com (Z.W.); hanhaiming@caas.cn (H.H.); zhoushenghui@caas.cn (S.Z.); guobaojin1991@126.com (B.G.); zhangjinpeng@caas.cn (J.Z.); yangxinming@caas.cn (X.Y.); lixiuquan@caas.cn (X.L.); 2 State Key Laboratory for Biology of Plant Diseases and Insect Pests, Institute of Plant Protection, Chinese Academy of Agricultural Sciences, Beijing 100193, China; tgliu@ippcaas.cn

**Keywords:** wheat, *Agropyron cristatum*, wheat–*A. cristatum* 2P addition line, resistance gene to leaf rust, gene expression

## Abstract

Wheat leaf rust (caused by *Puccinia triticina* Erikss.) is among the major diseases of common wheat. The lack of resistance genes to leaf rust has limited the development of wheat cultivars. Wheat–*Agropyron cristatum* (*A. cristatum*) 2P addition line II-9-3 has been shown to provide broad-spectrum immunity to leaf rust. To identify the specific *A. cristatum* resistance genes and related regulatory pathways in II-9-3, we conducted a comparative transcriptome analysis of inoculated and uninoculated leaves of the resistant addition line II-9-3 and the susceptible cultivar Fukuhokomugi (Fukuho). The results showed that there were 66 *A. cristatum* differentially expressed genes (DEGs) and 1389 wheat DEGs in II-9-3 during *P. triticina* infection. Kyoto Encyclopedia of Genes and Genomes (KEGG) pathway enrichment and gene set enrichment analysis (GSEA) revealed that the DEGs of II-9-3 were associated with plant–pathogen interaction, MAPK signaling pathway–plant, plant hormone signal transduction, glutathione metabolism, and phenylpropanoid biosynthesis. Furthermore, many defense-related *A. cristatum* genes, such as two NLR genes, seven receptor kinase-encoding genes, and four transcription factor-encoding genes, were identified. Our results indicated that the key step of resistance to leaf rust involves, firstly, the gene expression of chromosome 2P upstream of the immune pathway and, secondly, the effect of chromosome 2P on the co-expression of wheat genes in II-9-3. The disease resistance regulatory pathways and related genes in the addition line II-9-3 thus could play a critical role in the effective utilization of innovative resources for leaf rust resistance in wheat breeding.

## 1. Introduction

Wheat leaf rust caused by *Puccinia triticina* Erikss. is one of the main diseases hindering wheat production [1]. The life cycle of *P. triticina* consists of five spore stages. Urediniospores are formed on the wheat host, and they will develop into black teliospores on leaves at plant maturity. After the teliospores germinate, they produce basidiospores and are carried to the alternate hosts by the wind. Then, the basidiospores develop into pycniospores and aeciospores. When the aeciospores are spread to wheat hosts, and urediniospores are produced in the right environment, the life cycle is complete [1,2]. Leaf rust spreads urediniospores by airflow and can spread widely in a short period of time under suitable environmental conditions, resulting in severe yield reductions, up to 50% in severe cases [3]. Compared with chemical control, the cultivation of disease-resistant varieties is more economical and environmentally friendly and plays a key role in the control of wheat leaf rust [4,5,6]. At present, the leaf rust disease resistance genes *Lr1*, *Lr2a*, *Lr2c*, *Lr3*, *Lr16*, *Lr26*, *Lr11*, *Lr17*, *Lr10*, *Lr14a*, *Lr2b*, *Lr3bg*, *Lr14b*, *Lr32*, *Lr33* and *Lr50* have lost disease resistance to most *P. triticina* races due to environmental changes and variations in physiological races in China [7,8,9]. The discovery and utilization of new genes for resistance to leaf rust can overcome the epidemics of leaf rust caused by long-term and widely used resistance genes. The leaf rust resistance gene *Lr42* from *Aegilops tauschii* is widely used in wheat breeding programs, which is attributed to broad-spectrum leaf rust resistance [10]. Broad-spectrum leaf rust resistance genes have important value in wheat disease resistance breeding, but they are very scarce in wheat varieties [11]. Therefore, the continual discovery of new broad-spectrum leaf rust resistance genes is of great significance to wheat disease resistance breeding.

Wild relatives of wheat provide abundant genetic resources associated with high yield, disease resistance, and stress resistance for wheat, and the introduction of excellent genes of the relatives into cultivated wheat is beneficial to the improvement of wheat varieties [12,13]. Resistance genes such as *Yr9*, *Pm8*, *Lr26*, and *Sr31* of rye (*Secale cereale*) chromosome 1RS [14,15,16]; powdery mildew resistance genes *Pm21* and *Pm67* of *Haynaldia villosa* chromosome 6V and 1V [17,18,19]; Fusarium head blight resistance gene *Fhb7* of *Thinopyrum elongatum* [20]; stripe rust resistance gene *YrAS2388*; and leaf rust resistance gene *Lr42* of *Ae. tauschii* [10,21] have been successfully applied in wheat breeding. *Agropyron cristatum* (*A. cristatum*) (2*n* = 4*x* = 28, PPPP), a wild relative of wheat, has resistance to some major diseases, such as wheat rust, powdery mildew, and yellow dwarfing; thus, this species can be used as an excellent genetic source for wheat [22,23,24,25,26].

We identified and obtained a wheat–*A. cristatum* 2P disomic addition line II-9-3. Chromosome 2P of wheat–*A. cristatum* addition line II-9-3 carries genes that provide broad-spectrum immunity to leaf rust and high resistance to powdery mildew, according to genetic analysis [27]. The wheat–*A. cristatum* 2P disomic addition line II-9-3 is immune or nearly immune to 50 leaf rust races collected from 102 different locations in 13 provinces in China, so it carries broad-spectrum leaf rust resistance genes. The postulation of resistant genes showed that the leaf rust resistance gene of the addition line II-9-3 was a new and broad-spectrum resistance gene, which had a wider resistance spectrum than the total spectrum of the leaf rust resistance gene *Lr1*, *Lr2c*, *Lr3*, *Lr16*, *Lr26*, *Lr3ka, Lr11*, *Lr17*, and *Lr30* [28]. Therefore, we explored the excellent leaf rust resistance genes from *A. cristatum* chromosome 2P and analyzed the regulatory mechanism of the wheat–*A. cristatum* 2P addition line, given that wheat leaf rust resistance is highly important for wheat disease resistance breeding.

Transcriptome sequencing technology can quickly and comprehensively provide almost all the gene information of a species in a certain state, so it has become an important tool to study gene transcript levels and identify functional genes [29]. A large number of disease resistance genes have been mapped via RNA sequencing (RNA-seq). Li et al. performed RNA-seq analysis on the homozygous translocation line TA3465 of Chinese spring wheat–goatgrass and mapped the powdery mildew resistance gene *Pm66* to the short arm of 4Sl [30]. Wang et al. mapped the stripe rust resistance gene *YrZH22* to a 5.92 cM genetic range on wheat chromosome 4BL via bulked segregant RNA-seq (BSR-Seq) [31]. Using RNA-seq, Yang et al. identified and cloned the powdery mildew resistance gene *Pm40* [32]. Therefore, this study involved evaluating the transcriptome of inoculated and uninoculated leaves of a resistant addition line, II-9-3, and susceptible cultivar, Fukuhokomugi (Fukuho). The promising disease-responsive gene candidates were profiled, and the pathways involved were analyzed. It is expected that this work will provide a basis for further gene discovery and effective utilization in wheat leaf rust resistance breeding.

## 2. Results

### 2.1. Cytological Identification and P. Triticina Response to wheat–A. Cristatum 2P Addition Line II-9-3

**Figure 1:**
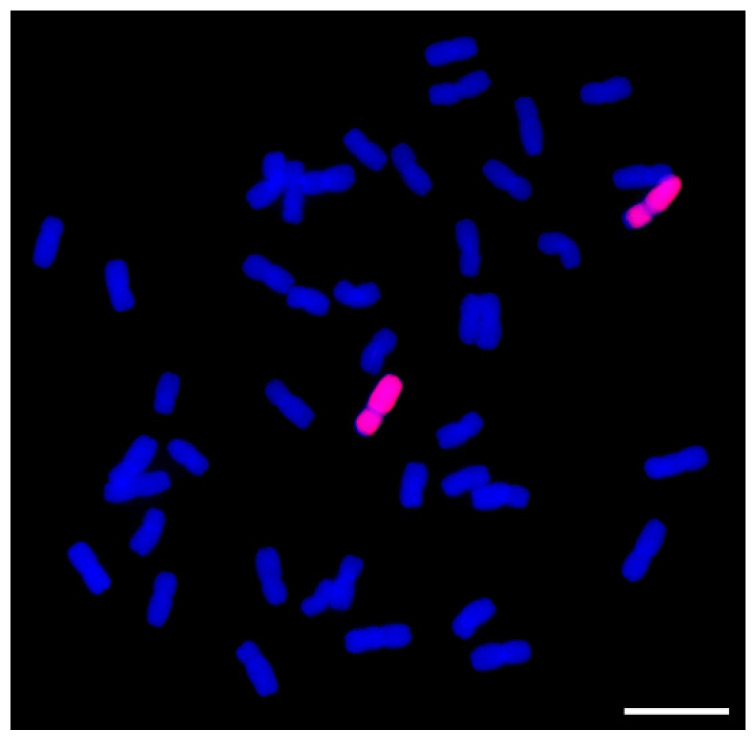
GISH results of wheat–*A. cristatum* 2P addition line II-9-3. Probe: *A. cristatum* genomic DNA. Block: wheat genomic DNA from Fukuho. Bar = 5 μm.

**Figure 2:**
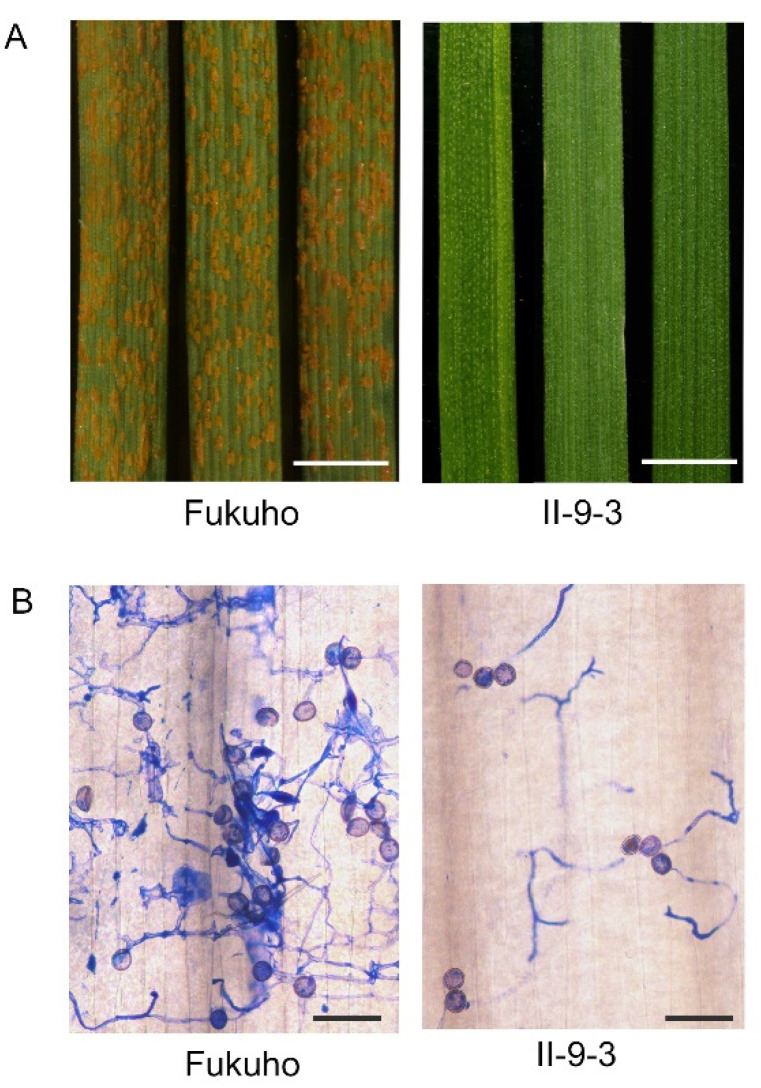
Response of wheat–*A. cristatum* 2P addition line II-9-3 and susceptible Fukuho to *P. triticina* race THT: (**A**) disease symptoms of leaves at 10 days after inoculation with *P. triticina* race THT (bar = 5 mm); (**B**) Coomassie brilliant blue staining of the leaves at 72 h post-inoculation (hpi) with THT to visualize spores (bar = 100 µm).

To confirm the stability of the addition chromosome 2P in II-9-3, II-9-3 was identified by genome in situ hybridization (GISH). In total, 10 II-9-3 seeds were randomly selected for GISH identification, and the results showed that the 10 individual plants of II-9-3 had 2 chromosomes from *A. cristatum* and 42 chromosomes from wheat (Figure 1). To assay the resistance of II-9-3 to *P. triticina* race THT, the addition line II-9-3 and the susceptible cultivar Fukuho were inoculated with physiological race THT at the seedling stage. Seedling responses to THT at 10 days post-inoculation THT indicated that II-9-3 was immune with IT = 0, whereas Fukuho was highly susceptible with IT = 3 (Figure 2A,B). Thus, chromosome 2P has at least one gene that provides moderate resistance to leaf rust.

### 2.2. RNA-Seq Quantity Analysis of the Addition Line II-9-3 and Susceptible Cultivar Fukuho

To understand the transcriptional regulation of disease-resistance-related genes in the addition line II-9-3, we used treated and nontreated leaves from the II-9-3 and Fukuho for RNA-seq. In total, 12 cDNA libraries comprising the II-9-3 and Fukuho inoculated groups (II-9-3_T, Fukuho_T) and the control group (II-9-3_CK, Fukuho_CK), with 3 biological replications, were used for each sample. In all, II-9-3 and Fukuho provided 343,794,274 and 348,383,084 clean reads after the quality filtering process. The Q20 and Q30 percentages of II-9-3 and Fukuho were greater than 96.55% and 91.73%, respectively (Table S1). At least 89.42% of the clean reads were mapped to the wheat reference genome (IWGSC RefSeq v2.1) and chromosome 2P of the *A. cristatum* genome. A total of 2500 *A. cristatum* genes and 46,754 wheat genes were detected among the 12 libraries. Subsequently, the correlation based on fragments per kilobase of exon per million fragments mapped (FPKM) among the 12 samples indicated high quality and reproducibility (Figure S1).

### 2.3. DEGs Related to Disease Resistance

**Figure 3:**
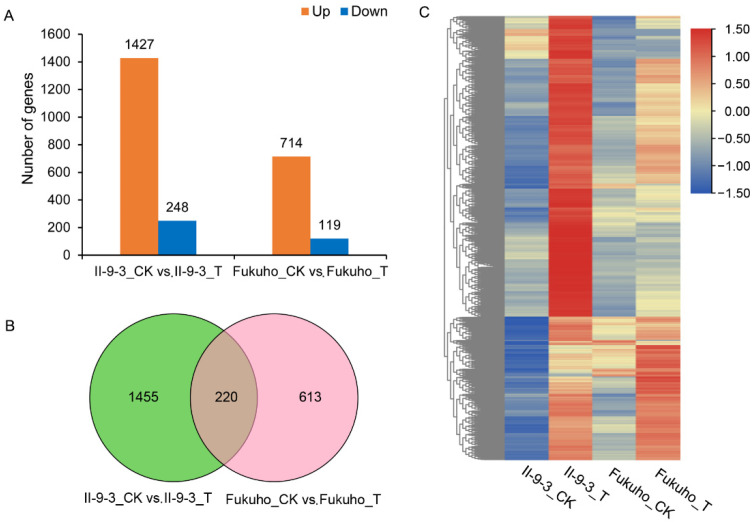
Analysis of DEGs of the addition line II-9-3 and Fukuho before and after inoculation: (**A**) Number of DEGs; (**B**) Venn diagram of DEGs before and after inoculation; (**C**) Heatmap of DEGs of each sample.

We adopted stringent criteria (|log2 (fold change) (log2FC)| > 1 and an adjusted *p* value < 0.05) to detect DEGs between the inoculated and uninoculated groups. A total of 1675 DEGs comprising 1427 upregulated genes (52 *A. cristatum* genes and 1375 wheat genes) and 248 downregulated genes (14 *A. cristatum* genes and 234 wheat genes) were detected in II-9-3_CK vs. II-9-3_T (Figure 3A). However, a total of 833 DEGs were identified in Fukuho, 119 of which were downregulated, and 714 of which were upregulated (Figure 3B,C). These results suggested that more genes were involved in the resistance to leaf rust in the II-9-3, and the expression of the 2P genes in II-9-3 might affect the gene expression of recipient wheat.

**Figure 4:**
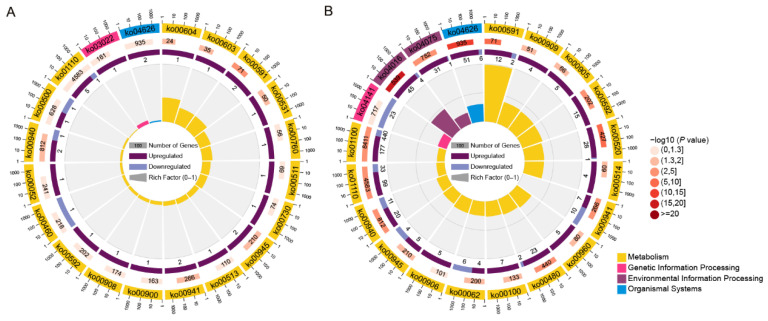
The top 20 KEGG enrichment results of DEGs in the addition line II-9-3: (**A**) KEGG enrichment of *A. cristatum* DEGs; (**B**) KEGG enrichment of wheat DEGs.

To further understand the biological pathways of resistance responses of II-9-3 to leaf rust, Kyoto Encyclopedia of Genes and Genomes (KEGG) pathways enrichment was mined for specific DEGs in II-9-3_CK vs. II-9-3_T (Figure 4, Table S3). The most represented pathway of the *A. cristatum* gene was the basal transcription factor pathway. The pathways in which *A. cristatum* and wheat genes were involved include plant–pathogen interaction, plant hormone signal transduction, flavonoid biosynthesis, phenylpropanoid biosynthesis, alpha-linolenic acid metabolism, and stilbenoid, diarylheptanoid, and gingerol biosynthesis. These results indicated that transcription factors of *A. cristatum* play an important role in activating the expression of genes related to disease resistance and lead to the broad-spectrum immunity of the addition line II-9-3.

**Figure 5:**
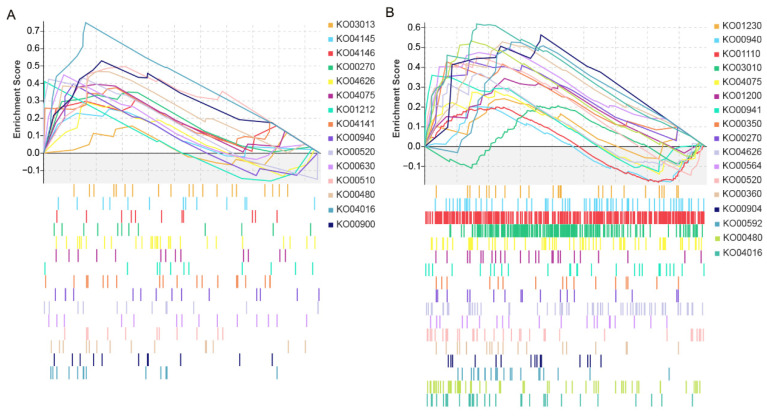
GSEA of genes in the addition line II-9-3: (**A**) GSEA of *A. cristatum* genes; (**B**) GSEA of wheat genes.

As KEGG enrichment focuses only on significant DEGs, the contribution of genes to disease resistance phenotypes cannot be determined. Thus, the special genes in II-9-3 after inoculation with *P. triticina* were examined using gene set enrichment analysis (GSEA) to identify the key pathways associated with disease-resistant gene expression (Figure 5, Table S4). This analysis revealed N-glycan biosynthesis, glyoxylate and dicarboxylate metabolism, and protein processing in the endoplasmic reticulum and peroxisome as the targets of specific gene sets of *A. cristatum*. Plant–pathogen interaction, MAPK signaling pathway–plant, plant hormone signal transduction, glutathione metabolism, phenylpropanoid biosynthesis, and amino sugar and nucleotide sugar metabolism were shared gene sets of *A. cristatum* and wheat. Both KEGG pathway enrichment analysis and GSEA indicated that the important biological processes associated with disease resistance in II-9-3 were plant–pathogen interaction, MAPK signaling pathway–plant, plant hormone signal transduction, glutathione metabolism, and phenylpropanoid biosynthesis. In addition, *A. cristatum* genes play important roles in transcription factors, N-glycan biosynthesis, glyoxylate and dicarboxylate metabolism, and protein processing in the endoplasmic reticulum and the peroxisome.

### 2.4. Identification of A. cristatum Genes Related to Plant Disease Resistance Pathways

**Figure 6:**
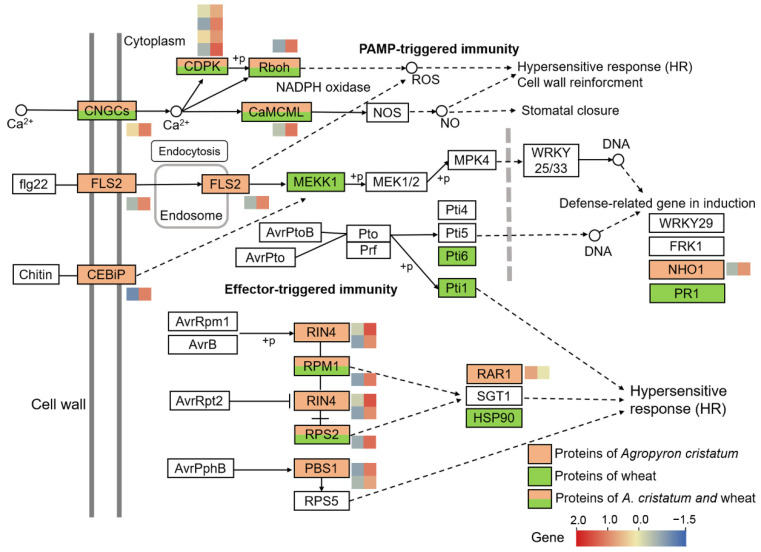
Schematic diagram of the plant*–*pathogen interaction pathway involving DEGs in the addition line II-9-3. Red boxes: proteins encoded by genes of *A.* *cristatum*; green boxes: proteins encoded by genes of wheat. Genes from *A. cristatum* encoding these enzymes as well as their expression are presented in the heatmap.

Plants initiate pathogen attacks via pattern-triggered immunity (PTI) and effector-triggered immunity (ETI), which are initiated by cell-surface-localized pattern-recognition receptors (PRRs) and intracellular nucleotide-binding domain leucine-rich repeat-containing receptors (NLRs), respectively [33]. In the addition line II-9-3, 25 *A. cristatum* genes and 70 wheat genes were enriched in the plant–pathogen interaction pathway. Although the number of *A. cristatum* genes from chromosome 2P was lower than that of wheat, the genes played a key role in the upstream of the PTI and ETI (Figure 6). An FLS2 homolog (*Ac2P01G432500*) with a serine/threonine protein kinase structure and a chitin receptor CEBIP homolog (*Ac2P01G18549*) were upregulated upstream of PTI. The upstream ETI pathway was annotated with two NLR genes (*Ac2P01G359200* and *Ac2P01G19996*), two serine/threonine protein kinases (*Ac2P01G277300* and *Ac2P01G19571*), and two RPM1-interacting proteins (*Ac2P01G28240* and *Ac2P01G19498*). However, wheat genes were annotated only in the downstream signaling pathways of PTI and ETI.

In addition, nine *A. cristatum* resistance-associated genes were identified in the MAPK signaling pathway–plant: four genes encoding serine/threonine protein kinases (*Ac2P01G327800, Ac2P01G18007, Ac2P01G19645* and *Ac2P01G581900*), four genes encoding chitin recognition proteins (*Ac2P01G590000, Ac2P01G591200*, *Ac2P01G591500* and *Ac2P01G581800*), and one gene encoding a mitogen-activated protein kinase (MAPK) (*Ac2P01G381300*). Two genes encoding glutathione transferase (GST) were identified in the glutathione metabolic pathway (*Ac2P01G563000* and *Ac2P01G18556*). Four genes encoding transcription factors were identified in the plant hormone signal transduction pathway: These genes included one bZIP transcription factor (*Ac2P01G498600*), two TGA transcription factors (*Ac2P01G498500* and *Ac2P01G20623*), and one BES1/BZR1 transcription factor (*Ac2P01G18927*). A universal transcription factor, TFIIB (*Ac2P01G20658*), was identified via the basic transcription factor pathway.

### 2.5. Verification of the Expression Patterns for Quantitative Real-Time PCR (qRT-PCR) of Disease Resistance-Related A. Cristatum Genes

**Figure 7:**
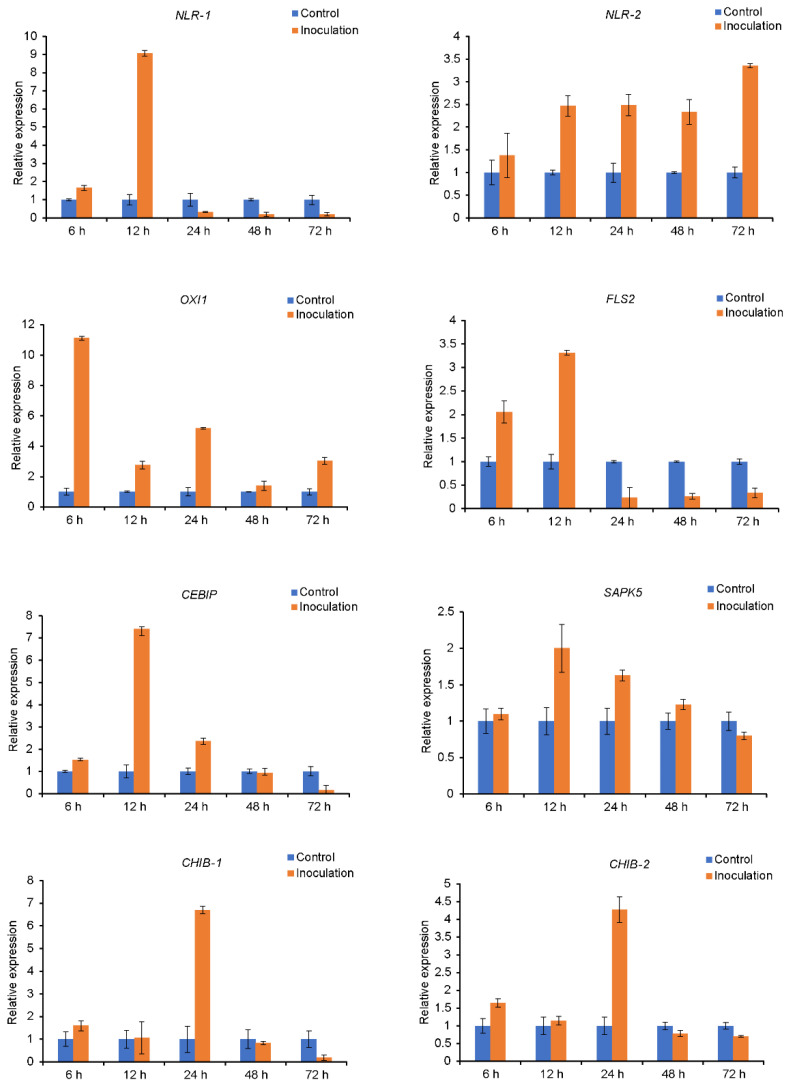
Expression patterns of disease resistance-related genes of the addition line II-9-3 at five time points after inoculation.

The expression patterns of eight *A. cristatum* genes related to disease resistance at five time points in the addition line II-9-3 were determined via qRT-PCR (Figure 7). At five different time points after inoculation, two NLR encoding transcripts showed differential expression patterns in II-9-3. The *NLR-1* gene showed the highest expression at 12 h and ultimately abruptly decreased 24 h after inoculation, whereas the expression of the *NLR-2* gene was significantly higher than that in the uninoculated leaves. Accordingly, the differences in the expression patterns of *NLR-1* and *NLR-2* were in accordance with the various roles of these genes. Genes associated with fungal cell wall chitin were upregulated in the early phase of infection. One gene encoding chitin-binding protein (CEBIP) exhibited the highest expression levels at 12 hpi, and two genes encoding chitin recognition enzyme (CHIB) showed the highest expression at 24 hpi. In addition, the expression levels of genes encoding the receptor kinases FLS2, OXI1, and SAPK5 peaked at 12 hpi and then decreased. The mean relative expression at the five time points indicated that the expression levels of the abovementioned eight genes were upregulated after rust inoculation. Their qPCR results were in accordance with the results of RNA-seq.

### 2.6. Chromosome 2P of the Addition line II-9-3 Influenced the Gene Expression Pattern of Recipient Wheat

**Figure 8:**
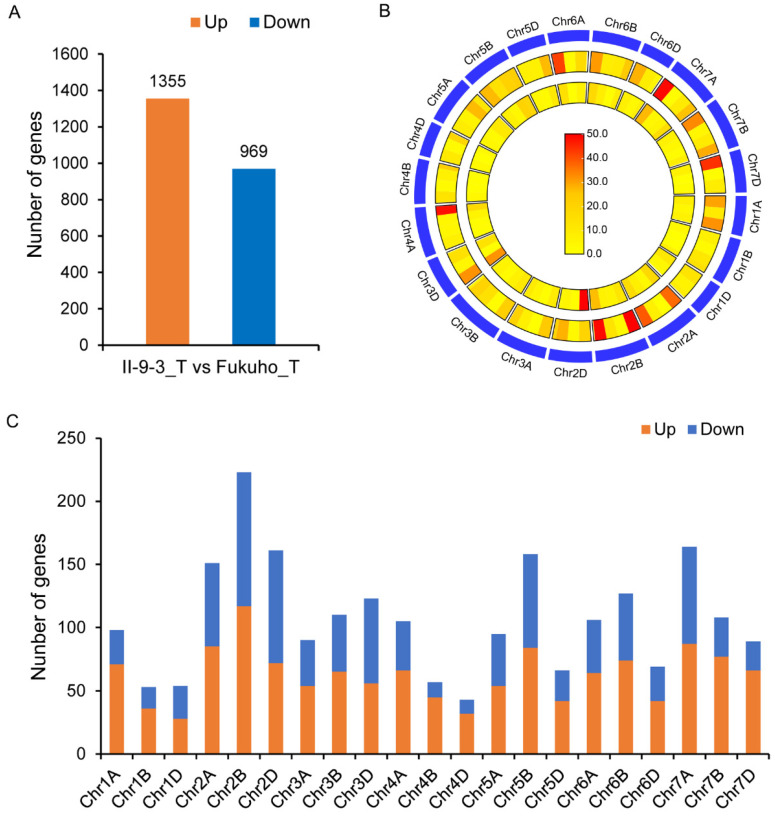
Wheat DEGs of II-9-3_T vs. Fukuho_T: (**A**) number of wheat DEGs of the addition line II-9-3; (**B**) heatmap of the gene density distribution of wheat DEGs. The inner circle represents the downregulated genes, and the outer circle represents the upregulated genes; (**C**) distribution of DEGs across the 21 wheat chromosomes.

The expression of genes from chromosome 2P of the addition line II-9-3 may also affect the gene expression of the recipient wheat. To further analyze the effects of chromosome 2P on the gene expression of wheat in the addition line II-9-3, trend clustering of wheat DEGs in each sample was carried out via FPKM. The results indicated that the expression patterns of a large number of wheat genes in II-9-3 were different from those in Fukuho (Figure S2). The expression levels of 640 wheat genes did not change in Fukuho but were significantly upregulated in II-9-3 after inoculation (Figure 2A,D). These results indicated that these genes are regulated by chromosome 2P. To further identify wheat genes expressed or inhibited specifically in II-9-3, the criteria were based on |log2FC| ≥ 4 and adjusted *p* value < 0.05 [34]. A total of 1355 wheat genes were upregulated in the inoculation group (II-9-3_T vs. Fukuho_T), and 969 wheat genes were downregulated (Figure 8A). These genes were mapped onto the chromosome of wheat genomes, and it was found that genes whose expression changed were more distributed at the chromosomal ends, among which chromosome 2B had the largest number of DEGs (Figure 8B,C). These results indicated that the wheat genes involved in resistance to leaf rust showed different expression patterns after inoculation with II-9-3 due to the influence of chromosome 2P.

## 3. Discussion

In the process of plant growth*,* development, and stress tolerance, significant changes in gene expression occur. With the development of sequencing technology and related methods, transcriptome analysis has been widely used to reveal the molecular regulatory mechanisms underlying plant disease responses [35,36]. Our study involved the use of the *P. triticina* resistant addition line II-9-3 and the susceptible cultivar Fukuho to explore the transcriptional changes after inoculation. Compared with the susceptible cultivar Fukuho, II-9-3 had more DEGs involved in the response to *P. triticina* race THT infection, and there were 1455 specific DEGs, namely, 66 *A. cristatum* genes and 1389 wheat genes. The two *A. cristatum* chromosomes in the addition line accounted for only 4.55% of the total 44 chromosomes, and there were relatively few DEGs in the addition line II-9-3. The genes of *A. cristatum* may be upstream of the regulatory pathway in response to II-9-3 disease resistance, which is upregulated at individual time points in response to *P. triticina*, and the statistical significance of the gene expression may have been affected by sample mixing.

KEGG enrichment analysis counted only genes that were significantly differentially expressed, while GSEA, from the perspective of the enrichment of gene sets, was not limited to the DEGs, so it was theoretically easier to identify some genes that had small changes in expression but played a role in biological functions [37]. GSEA has been widely used for gene functional analysis in plants [38]. In this study, both KEGG enrichment analysis and GSEA revealed the disease resistance pathways of II-9-3. Plant–pathogen interaction was the most critical pathway, and the MAPK signaling pathway–plant, plant hormone signal transduction, glutathione metabolism, and phenylpropanoid metabolism have also been suggested to play important roles in regulating plant–fungus interactions [39,40]. In the key plant–pathogen interaction pathways, the *A. cristatum* genes were annotated as functioning upstream of PTI and ETI, while the wheat genes were annotated as functioning downstream. Recent advances have shown that ETI intensity limits plant resistance, but the PTI component is an important feature of ETI [41,42]. The results described above demonstrated that the key role of the addition line II-9-3 in the immunity of leaf rust first involves the upstream expression of genes from *A. cristatum*.

Receptor-like kinases (RLKs) and receptor-like proteins (RLPs) serve as PRRs to recognize danger signals of pathogens in plants [41]. The LRR receptor-like serine/threonine protein kinase gene is a PRR gene, which plays an important role in PTI [43]. A large number of receptor kinases in crops are related to disease resistance regulation; for example, the *LRK10* gene is closely linked to the wheat leaf rust resistance gene *Lr10* [44]. *Stpk-V,* a key member of the powdery mildew resistance gene *Pm21*, encodes serine/threonine protein kinases, and the wall-associated receptor kinase gene *OsWAK1* also plays an important role against rice blast fungus [45,46]. In this study, six *A. cristatum* receptor kinase genes were identified in the interaction of II-9-3 inoculated with *P. triticina*. ETI is activated by pathogen-effector proteins via NLRs [47]. The leaf rust disease resistance genes *Lr1*, *Lr10*, *Lr13*, *Lr21*, *Lr22a*, and *Lr42* [10,48,49,50,51,52,53] and the powdery mildew resistance gene *Pm21* are NLR genes [17,18]. Two upregulated NLR genes were identified, which had different expression patterns in the addition line II-9-3. Their role in disease resistance regulation needs to be verified via further experiments. In addition, the gene coding for GST was shown to play an important role in *Th. elongatum* [20]. Two *A. cristatum* GST genes were identified in this study, which may play an important role in disease resistance.

The expression of *A. cristatum* genes in the addition line II-9-3 resulted in more substantial differential changes in the expression of wheat genes. A total of 1355 wheat genes were upregulated, and 969 wheat genes were downregulated in II-9-3_T vs. Fukuho_T, which accounted for 5.93% of the total expressed wheat genes. The effect of chromosome 2P on Fukuho gene expression was higher than that of Elodie et al. [54]. Elodie et al. identified 960 DEGs between the Chinese spring wheat and Chinese spring wheat–barley 7H addition line, namely, 509 downregulated genes and 451 upregulated genes, accounting for 2.7% of the total expressed genes in wheat. However, chromosome 2P of the addition line II-9-3 affected only the transcriptional activity of part of some genes, while most genes maintained their regular transcriptional activity.

## 4. Materials and Methods

### 4.1. Experimental Materials and Leaf Rust Inoculation

The plant materials in this study included *A. cristatum* accession Z559 (2*n* = 4*x* = 28, PPPP), the common wheat cultivar Fukuho (2*n* = 6*x* = 42, AABBDD), and wheat–*A. cristatum* 2P addition line II-9-3 (2*n* = 42 + 2). All the materials were provided by the Wheat Resources Laboratory, Institute of Crop Science, Chinese Academy of Agricultural Sciences.

The wheat–*A. cristatum* 2P addition line II-9-3 and common wheat cultivar Fukuho were planted in a greenhouse under 16 h of light and 8 h of darkness. When the seedlings were at the one-leaf stage, the spray method was used for inoculation [22,28]. The urediniospores (*P. triticina* isolate THT, collected from Beijing, China) suspension used was prepared by resuspending spore powder in light mineral oil (Chevron Phillips Chemical, Houston, TX, USA); the spore suspension was sprayed evenly on the wheat leaves, and the uninoculated control group was evenly sprayed with light mineral oil only. After the volatilization of mineral oil, a 0.05% Tween-20 (Sigma-Aldrich, St. Louis, MO, USA) aqueous solution was sprayed evenly onto the leaves of wheat. Then, plants were subjected to 18 °C in the dark, under 100% relative humidity for 24 h, and then moved to a greenhouse at 18–25 °C. A 0–4 scale was used to record the plants’ response to leaf rust infection according to the methods of Roelfs et al. [55].

### 4.2. GISH for II-9-3 Identification

GISH was performed as described by Liu et al. [56]. Genomic DNA of *A*. *cristatum* was used as a probe, and the common wheat cultivar Fukuho was used as a blocker to detect *A. cristatum* chromosome fragments in the background of wheat. Fluorescence signals were captured using an Olympus Zeiss AX10 (Olympus Corporation, Tokyo, Japan) microscope equipped with a charge-coupled device (CCD) (Diagnostic Institute, Inc., Sterling Height, MI, USA) camera, and in conjunction with Isis analyzed software (Metasystems GmbH, Altlussheim, Germany).

### 4.3. Histochemical Observations

Leaves were cut into 2 cm segments and then fixed in ethanol: glacial acetic acid (*v*/*v* = 1:1) (Aladdin Biochemical Technology, Shanghai, China) for 24 h. Staining with Coomassie brilliant blue was performed after rinsing twice with deionized water. The number of fungal colonies was counted under an Olympus BX53F microscope (Olympus Corporation, Tokyo, Japan).

### 4.4. RNA Sequencing and Transcriptome Analysis

Leaf tissues were collected from inoculated and uninoculated plants at five time points (6, 12, 24, 48, and 72 hpi) for three biological replicates. The leaf samples were instantly frozen and ground into a fine powder in liquid nitrogen in a mortar and pestle to isolate RNA using an RNA Isolation Kit (Zoman Biotechnology, Beijing, China). The RNA integrity was assessed using the RNA Nano 6000 Assay Kit of the Bioanalyzer 2100 system (Agilent Technologies, Palo Alto, CA, USA). RNA-seq of the resulting 12 libraries was conducted on an Illumina HiSeq 2500 sequencing platform at Novogene (Novogene, Tianjin, China). RNA-seq data for this study have been deposited in the NCBI database under BioProject accession number PRJNA838495.

The mapping of 12 sample reads to the wheat genome (IWGSC RefSeq v2.1) and chromosome 2P of the *A. cristatum* Z559 genome (data to be published in our laboratory) was performed with the help of HISAT2 software with the default parameters [57]. The new transcripts were assembled with StringTie with default settings [58]. Differential expression analysis of the two conditions was performed using DESeq2 [59]. KEGG functional annotation of differentially expressed genes was implemented by Eggnog-Mapper (accessed on 22 January 2022, http://eggnog-mapper.embl.de/). KEGG enrichment analysis was performed using the OmicShare tools (accessed on 9 March 2022, https://www.om-icshare.com/tools), and TBtools software (v1.075) was used to construct heatmaps and Venn diagrams [60].

### 4.5. Quantitative Real-Time PCR

To verify the RNA-seq data, eight *A. cristatum* genes were selected for qRT-PCR. The qRT-PCR primers were designed using Primer 5 (Table S2). Total RNA was reverse transcribed into cDNA using a HiScript III 1st Strand cDNA Synthesis Kit (Vazyme Biotech, Nanjing, China). On an ABI StepOne Plus Real-Time PCR System (Applied Biosystems, Carlsbad, CA, USA), RT-qPCR was performed using 2–Realstar Fast SYBR qPCR Mix (Genstar Technologies, Beijing, China). The *ARF* gene was used as a housekeeping gene [61]. Three technical replicates were included for each biological sample, and relative quantifications were calculated using the comparative 2^−ΔΔCT^ method.

## 5. Conclusions

Overall, our comprehensive analysis of the transcriptome data provided insights into the gene expression profile and disease resistance processes of the addition line II-9-3. II-9-3 might protect against rust infection mainly through genes associated with plant–pathogen interaction, the MAPK signaling pathway–plant, plant hormone signal transduction, glutathione metabolism, and phenylpropanoid biosynthesis. We further identified genes that encode receptor protein kinases, NLRs, and transcription factors from *A. cristatum* involved in key immune regulatory pathways. The present findings provide support for the resistance mechanism of the addition line II-9-3 and set the stage for the exploration, cloning, and functional research of leaf rust resistance genes from chromosome 2P of *A. cristatum*.

## Data Availability

The data for this study have been deposited in the NCBI database under BioProject accession number PRJNA838495.

## Supplementary Materials

The following supporting information can be downloaded at: https://www.mdpi.com/article/10.3390/ijms23137370/s1.
