# Supplementary material for: Comparative Transcriptome Analysis Reveals the Gene Expression and Regulatory Characteristics of Broad-Spectrum Immunity to Leaf Rust in a Wheat–Agropyron cristatum 2P Addition Line
Source: Int J Mol Sci. 2022 Jul 1;23(13):7370. doi: 10.3390/ijms23137370 (PMC9266861; doi:10.3390/ijms23137370)
Supplement: Supplementary file 1 [file ijms-23-07370-s001.zip › Supplementary files/Figure S1.pdf]

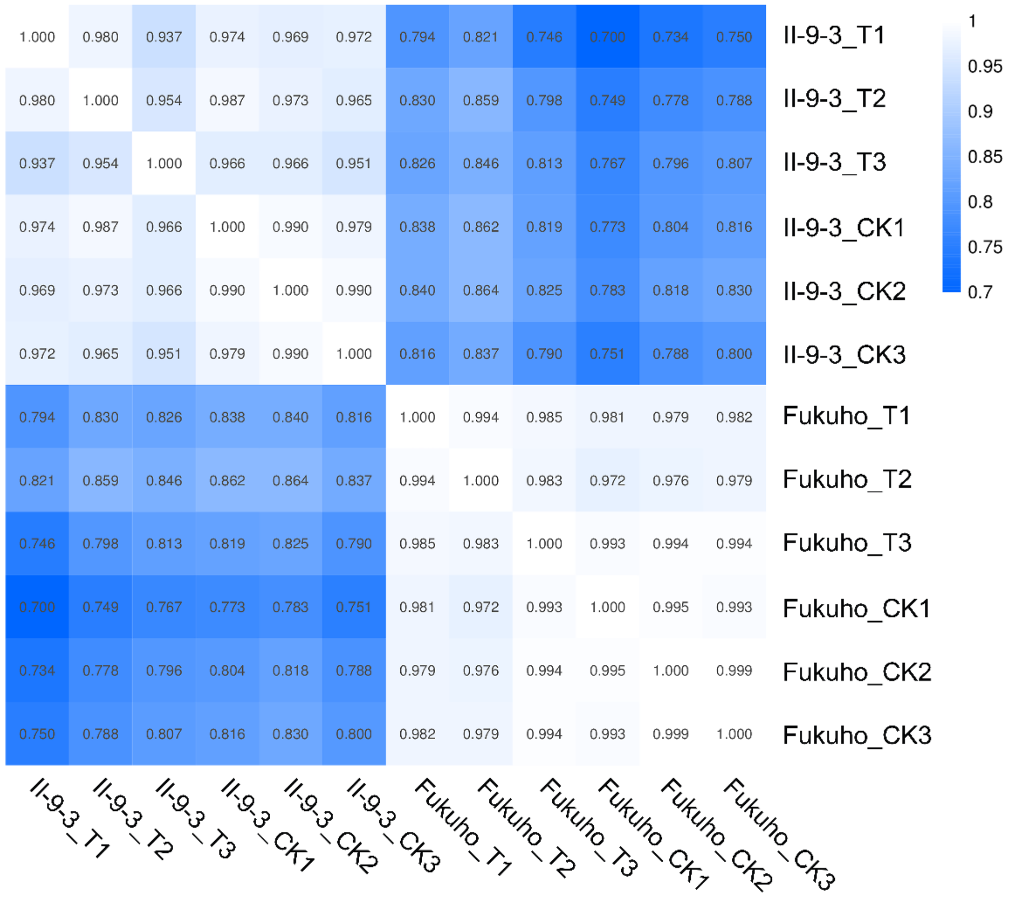

Figure S1. Correlation coefficients for each sample of addition line II-9-3 and recipient wheat Fukuho
